# Effectiveness of combined pregabalin and celecoxib for treatment of acute postoperative pain: A meta-analysis and systematic review

**DOI:** 10.1097/MD.0000000000032080

**Published:** 2022-12-30

**Authors:** Jing-Mei Ni, Xuan Zhu, Ping Wang

**Affiliations:** a Department of Anesthesiology, Sir Run Run Shaw Hospital, School of Medicine, Zhejiang University, Hangzhou, China.

**Keywords:** combined pregabalin and celecoxib, meta-analysis, postoperative pain

## Abstract

To estimate the acute analgesic efficacy of combined Pregabalin and Celecoxib after operation *via* a systematic review and meta-analysis.

**Methods:**

Studies for inclusion were randomized controlled trials, reporting on relevant outcomes (0–6 hours, 24 hours, 7 days pain scores) with treatment with combined Pregabalin and Celecoxib.

**Results:**

The pooled results from meta-analysis demonstrated that compared with placebo, combined Pregabalin and Celecoxib reduced pain scores at 0 to 6 hours in 3 articles, 24 hours in 5 articles, 7 days in 2 articles (standard mean difference [SMD], −3.10 at 0–6 hours, −2.80 at 24 hours, −1.32 at 7 days, respectively). Combined Pregabalin and Celecoxib could significantly reduce the postoperative narcotic consumption in 3 studies (SMD, −1.99 at 36 hour).

**Discussion:**

This work suggested that combined Pregabalin and Celecoxib were efficacious in reduction of postoperative pain and narcotic requirements after surgery, whereas more trials are needed to further identify the efficacy of combined Pregabalin and Celecoxib in the management of acute postoperative pain.

## 1. Introduction

Plenty of studies have revealed that acute post-operative pain occurred in approximately 80% of patients after surgery. Both mild and severe postoperative pain have negative influences on the lung and cardiovascular system. Additionally, it also delayed movement and reduced bladder and intestinal motility especially in the early days after surgery. Despite existing acquaintance in pathophysiology of pain and pharmacology of analgesics, as well as the development of strategies techniques in pain control, postoperative pain remains a major issue of concern in patient care.^[1]^

Multimodal analgesia with various pharmacologic agents provided efficiently adequate pain management, accompany with improvements in functional outcome, earlier ambulation, earlier discharge, and reduction of chronic pain. Although opioids play an important component in postoperative pain management, its’ side effects cannot be ignored.^[2]^ Hence, the multimodal analgesic approach has been recommended for the management of acute postoperative pain.^[3]^

Cyclooxygenase (COX)-2 specific nonsteroidal anti-inflammatory drugs (NSAIDs) and alpha 2-delta subunits calcium channel ligands (gabapentin and pregabalin) are 2 mechanistically different types of analgesics that initiated effect after various of surgical procedures.^[4]^ Both alpha 2-delta subunits calcium channel ligands and NSAIDs can interact synergistically or additively to reverse hyperalgesia relating to peripheral inflammation. Combination of Celecoxib and Pregabalin may demonstrate superior analgesic efficacy than either drug alone and cause fewer side effects.

Several studies have evaluated the efficacy of administering pregabalin with a NSAID for postsurgical analgesia, such as spinal fusion surgery,^[5–7]^ percutaneous endoscopic lumbar discectomy,^[8]^ total hip arthroplasty,^[9]^ maxillomandibular advancement surgery,^[10]^ laparoscopic cholecystectomy^[11]^ and total knee arthroplasty.^[12]^

The aim of this meta-analysis was to evaluate the analgesic efficacy of combining using Pregabalin and Celecoxib in reducing postsurgical pain, such as visual analogue scale (VAS) pain scores 0 to 6 hours, 24 hours, 7 days after surgery, expecting to provide a useful reference in perioperative care after various surgical. It is a key contributor to accelerate in the way to recovery. Reducing postoperative pain improves the quality of life and satisfaction of patients.

## 2. Materials and Methods

This review was performed according to the Preferred Reporting Items for Systematic Reviews and Meta-Analyses guidelines for reporting meta-analyses. Approval by ethics committee or written consent were not required for meta-analysis. Before this meta-analysis, all cited articles published within at least the abstract in English. Meanwhile all authors agreed on the inclusion and exclusion criteria and offered at least 1 of the major data.

All incorporated studies in the meta-analysis were randomized, double-blinded, controlled trials (RCTs). All research subjects were postoperative patients (age > 18 years) with no significant abnormalities in blood count, liver or kidney function. In addition, all referred to pain effects about intervention or treatment with combination of Pregabalin and Celecoxib (Patients in the test group were treated with Pregabalin combined with Celecoxib; patients in the control group were treated with pregabalin or celecoxib.). These studies had to present data for at least 1 of our prespecified variables, including 0 to 6 hours, 24 hours or 7 days postsurgical pain reactions.

**Figure 1.**
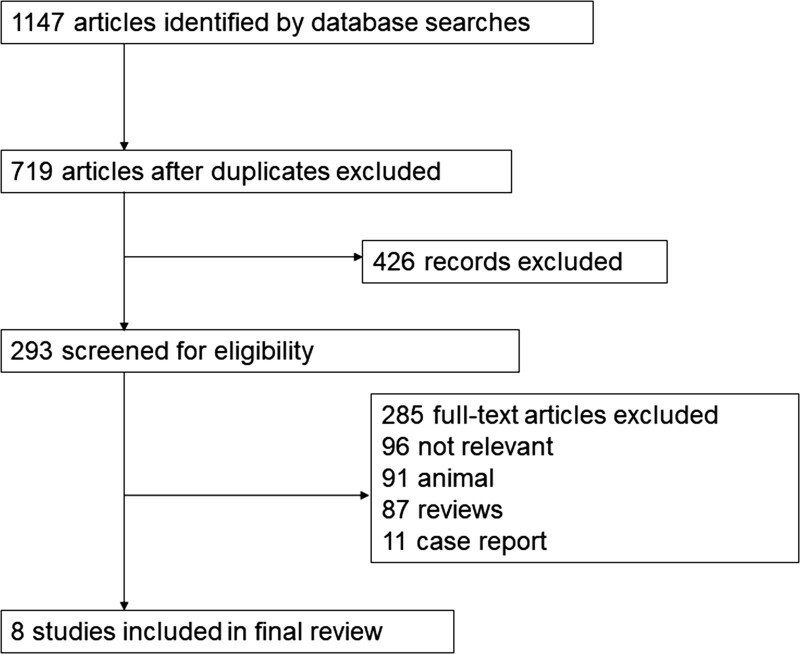
Flow diagram of screened, excluded, and analyzed publications. RCTs = randomized controlled trials.

We searched relevant literatures from initiation to May 2020 using Medline, Embase, Scopus, and Cochrane electronic databases with no limitation on publication year or language. In this study, literature retrieval was conducted using the following search terms individually or in combination: “Pregabalin,” “celecoxib,” “Postoperative Pain.” Studies (8 trials) for inclusion were randomized controlled trials, reporting on relevant outcomes (0–6 hours, 24 hours, 7 days pain scores) with treatment of combined Pregabalin and Celecoxib. All included studies could be considered to be of high quality in methodology (Fig. 1). Random effect model was used in our meta-analysis, and standard mean difference (SMD) as the pooled estimate. Two researchers independently read and selected the titles and abstracts of all retrieved articles to determine whether the articles contained potential data relevant to this study. In case of disagreement, the other author reviewed and made the final decision.

Information was extracted from each included trial (including country, age, sample size, and type of surgery) (Table 1). For continuous outcomes, including morphine consumption pain scores and patient satisfaction after procedure, we pooled study results using SMD. The SMD calculation required mean value and standard deviation from each group. Differences in means were considered significant at a *P *< .05. We used visual inspection of the forest plots to investigate the possibility of statistical heterogeneity, and the χ^2^ statistic. We did the analyses using Stata version 11 (StataCorp, Texas, TX). Data was analyzed using Comprehensive Meta-Analysis software (version 2.2.064, Englewood, NJ).

**Table 1:** Main characteristics of included trials.

id	Author	Surgery type	Study period	Country	Sample size	Age (yrs)	Follow-up (d)	Intervention	Complication
2006	Scott S. Reuben et al	Spinal fusion surgery	July 2004 and October 2005	America	80	>=18	24 h	Pre 1 h:400 mg/150 mg, post 12 h:200 mg/150 mg	Nausea/sedation
2013	Nicole ME Carmichael et al	Total hip arthroplasty	March 2008 and March 2009	Canada	80	18–80	6 wk	2 wk pre (75/100BID) 3 wk post discharge	Sedation dizziness blurred vision
2014	Joseph E. Cillo Jr. et al	Maxillomandibular advancement surgery	2 yrs	America	20	<60	36 h	1 h pre:150/400 mg	/
2016	Sang-Il Kim et al	Lumbar fusion surgery	May 2012 and October 2013	Korea	80	67.9 ± 7.6 66.3 ± 10.0	7d	1 h pre 75/200 mg; POST75BID/200QD	/
2016(2)	Usha Gurunathan1 et al	Laparoscopic cholecystectomy	July 2012 and July 2014	Australia	98	18–70	24h	1 h pre:150/400 mg	Sedation and nausea/vomiting
2017	Zhuhai LI et al	Percutaneous endoscopic lumbar discectomy	Between January and June 2014	China	81	18–65	3months	3 d pre: 75/200BID; pre 1 h 150; 10 d 150TId200BID;11–14d75TID/200BID	Sedation dizziness
2018	AndriM. T. Lubis et al	Total knee arthroplasty	July 2015 and December 2016	Indonesia	20	55–80	72h	1 h pre: 150/400 mg	/
2019	Nguyen Trung Kien1 et al	Lumbar spine surgery	March to September 2017	Vietnam	60	44.93 ± 10.26 48.23 ± 11.88	48h	2 h pre150/200	Sedation nausea/vomiting

## 3. Results

### 3.1. Postoperative narcotic requirements

**Figure 2.**
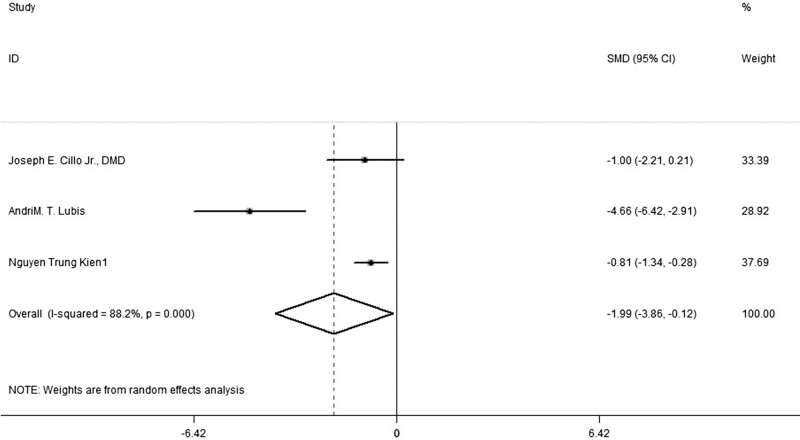
Forest plot of meta-analysis: 36 h morphine consumption (mg) in patients receiving combined Celecoxib and Pregabalin. CI = confidence interval.

Morphine was used as the analgesic medication in 3 trials. The quantitative data could be extracted from all 3 trials. The overall pooled results from meta-analysis demonstrated that compared with placebo, combined using of Pregabalin and Celecoxib for analgesia could significantly reduce the postoperative narcotic consumption at 36 hours (SMD, −1.99, 95% confidence interval [CI]: −3.86, −0.12, *P *= .000, random-effect model; Fig. 2).

### 3.2. Postoperative pain score at 0 to 6 hours

**Figure 3.**
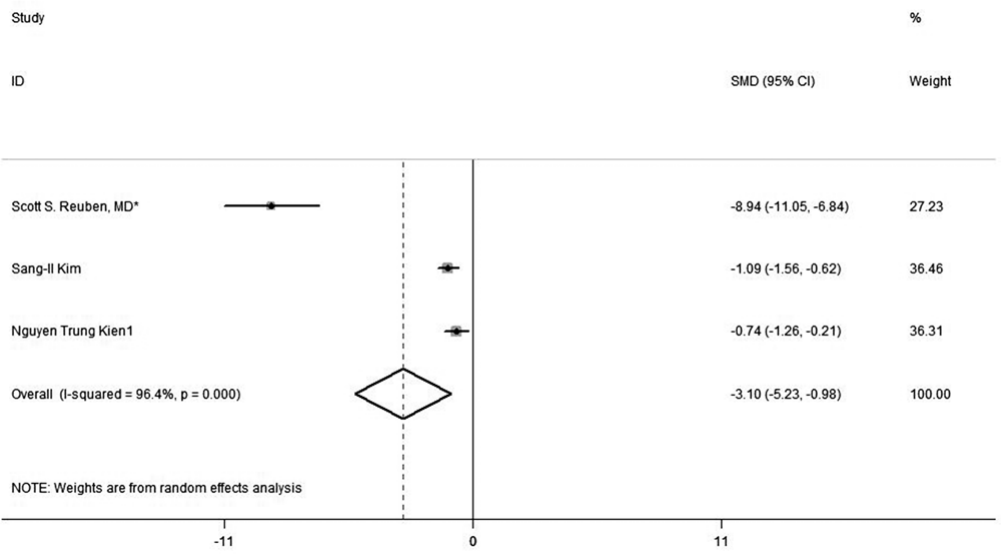
Forest plot of meta-analysis: VAS of postoperative pain intensity at 0 to 6 h in patients receiving combined Celecoxib and Pregabalin. CI = confidence interval, VAS = visual analogue scale.

This outcome was reported in 3 trials. The overall pooled results from meta-analysis demonstrated that compared with placebo, preemptive analgesia of combined Celecoxib and Pregabalin could significantly reduce the postoperative pain score at 0 to 6 hours. (SMD, −3.1, 95% CI: −5.23, −0.98, *P *= .000, random-effect model; Fig. 3).

### 3.3. Postoperative pain score at 24 hours

**Figure 4.**
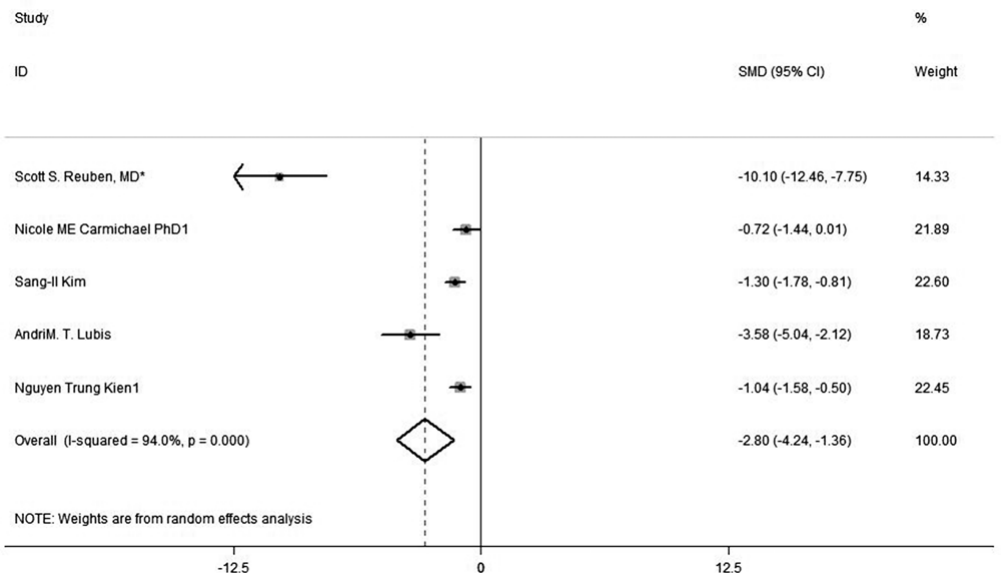
Forest plot of meta-analysis: VAS of postoperative pain intensity at 24 h in patients receiving combined Celecoxib and Pregabalin. CI = confidence interval, VAS = visual analogue scale.

**Figure 5.**
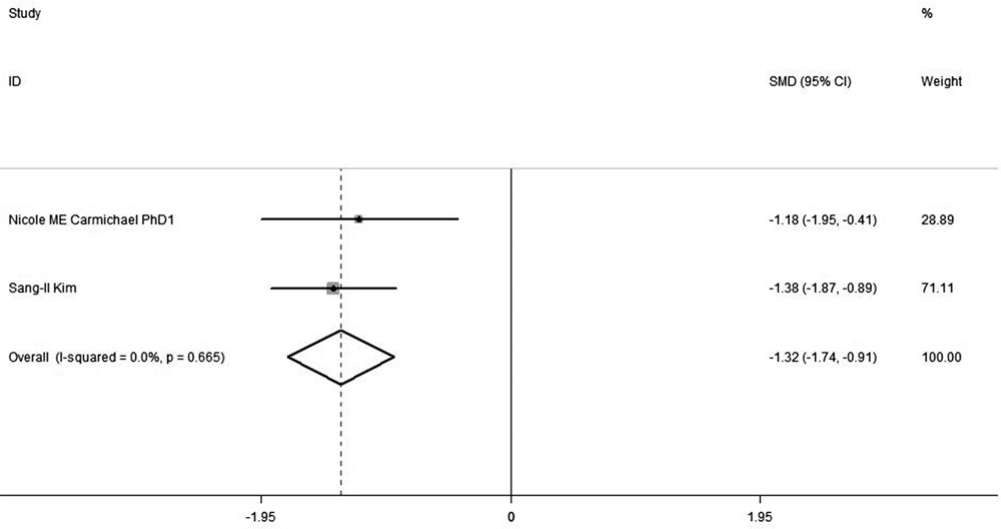
Forest plot of meta-analysis: VAS of postoperative pain intensity at 7 d in patients receiving combined Celecoxib and Pregabalin. CI = confidence interval, VAS = visual analogue scale.

The summarized results (revealed in 5 trials) in the meta-analysis showed that analgesic properties of combined Celecoxib and Pregabalin obviously decreased the post-procedure pain score at 24 hours with respect to placebo (SMD, −2.8, 95% CI: −4.24, −1.36, *P *= .000, random-effect model; Fig. 4). And subgroup analyses found that the combination exhibited lower analgesia points at 24 hours in contrast with single administration of Celecoxib (SMD, −0.89, 95% CI: −1.32, −0.47, *P *= .338; Fig. 5).

### 3.4. Postoperative pain score at 7 days

**Figure 6.**
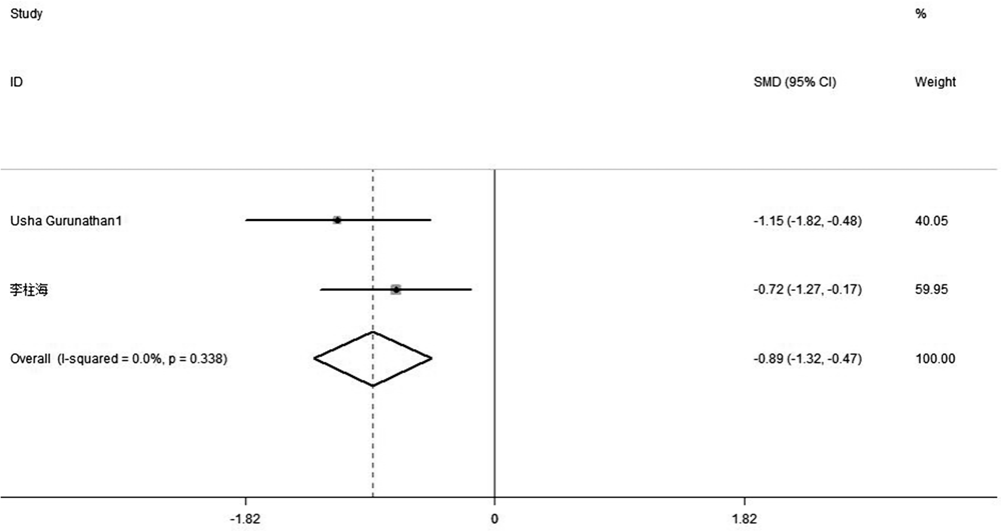
Forest plot of meta-analysis: VAS of postoperative pain intensity at 24 h in patients receiving combined Celecoxib and Pregabalin. CI = confidence interval, VAS = visual analogue scale.

Findings from meta-analyses (in 2 trials) uncovered that the analgesic score with utilization of Celecoxib and Pregabalin combination was notably lower than placebo administration at 7 d over procedure (SMD, −1.32,95% CI: −1.74, −0.91, *P *= .0665, random-effect model, Fig. 6).

## 4. Discussion

Multimodal analgesia, which means different mechanisms in improving pain relief and side effects from 2 or more analgesics and modalities, is increasingly popular in recent years. In 2016, the American Pain Society raise a suggestion that multimodal therapies could be performed in pre-operative period.^[13]^ A meta-analysis including 74 studies rendered that analgesic performances and adverse effects rate of Pregabalin are not equal in different procedure categories.

Preemptive multimodal (celecoxib, pregabalin, extended-release oxycodone, and acetaminophen) analgesic protocol have been done with significantly lower VAS in the multimodal analgesia group at all time points within 7 postoperative days.^[6]^ Previous studies elucidated that Celecoxib and Pregabalin were effective in moderating low-back pain. The combination of Celecoxib and Pregabalin was more valid than either monotherapy in patients with chronic low-back pain.^[14]^

Here, we reviewed related literature systematically and make a comprehensive understanding about the post-procedure pain management efficacy of these pharmaco-therapies. Due to the variety of drugs, regimen, and usage, the random effect model was chosen as the pooling method for this meta-analysis. Random effect model, a variation on the inverse-variance method, is to incorporate an assumption that different studies assessed different but related intervention.^[15]^ That generated a random-effects meta-analysis, the simplest version of which was termed to the DerSimonian and Laird method.^[16]^ Subgroup analysis was simultaneously executed for further analysis of each therapy effectiveness.

The pooled results suggested that combined Celecoxib and Pregabalin worked for pain alleviation at 0 to 6 hours, 24 hours, 7 days after surgery, concomitantly significantly reduced narcotics consumptions within low occurrence of side effects. However, more trials were needed to further evaluate its effectiveness. Pregabalin and Celecoxib could be well tolerated in our included trials.

This study was the first systematic review about the post-operation pain management regarding combined Celecoxib and Pregabalin. Nonetheless, there were still limitations, first of which was the small relatively sample that may lead to underestimation or overestimation. In addition, both pain score scale and narcotics dosage in each trial was not completely the same, which was the main reason that we choose the random effect model as the pooling method, and SMD as estimate. Finally, some trials did not manifest valuable information, which made it difficult to make full use of our included trials.

## 5. Conclusion

This work suggested that combined Celecoxib and Pregabalin were efficacious in reduction of postoperative pain and narcotic requirements after surgery. More trials are needed to further assess the efficacy of combined Celecoxib and Pregabalin in the management of postoperative pain, which could be considered as the option of multimodal analgesia.

## Author contributions

**Conceptualization:** Ping Wang.

**Data curation:** Jing-Mei Ni, Xuan Zhu, Ping Wang.

**Investigation:** Ping Wang.

**Methodology:** Jing-Mei Ni, Ping Wang.

**Writing – original draft:** Jing-Mei Ni, Xuan Zhu.

**Writing – review & editing:** Ping Wang.
